# Observations Tending to Show How Far Life Can Be Identified with Heat; with Some Strictures on Mr. Hunter's Experiments Relative to His Theory of Life

**Published:** 1821-09

**Authors:** James Down

**Affiliations:** Surgeon.


### Original Communications, Select Observations, etc.

---

### *Observations tending to show how far Life can be identified with Heat; with some Strictures on* Mr. HUNTER's *Experiments relative to his Theory of Life.* By JAMES DOWN, Surgeon.

"When once men take up with the opinions of others, they no longer improve the *science;* but servilely bestow their talents in adorning and defending some particular authors."—BACON.

IT might have been hoped, at this advanced period of science, that we should not have heard *vitality* identified with heat: but this notion has again been lately brought before the public by a man of science, who is in every way capable of instituting researches calculated to prove or disprove the inference. The conclusion here alluded to was stated by Mr. Brodie, in a Lecture delivered at the Royal College of Surgeons; wherein he said that, if a thermometer is placed in the trunk of a tree, the mercury will be raised two or three degrees above the freezing point, although the air surrounding the tree may be some degrees below it. The inference which he draws is this,—that it is the vitality of the tree which raises the mercury of the thermometer; in which he agrees with Mr. Hunter. How Mr. Hunter came to mistake this result for an effect of vitality, may be pretty easily accounted for. I shall not, however, enter into the particulars, but state an experiment which was instituted to prove the truth or error of the inference drawn by him, and from thence to inquire what cause he had for confounding heat with vitality.

On the 3d January, 1821, I pierced a hole into a young poplar-tree, obliquely downwards, from three to four inches in depth, just big enough to admit the bulb of a thermometer, and into which such an instrument was placed. I then drove a nail into the tree, to which I suspended another thermometer. I examined them in about an hour afterwards, and found them both standing at 31°, or one degree below the freezing point.

This experiment, it may be said, does not touch the correctness of Mr. Hunter's observation: no, nor is it supposed to do so; but rather his inference, that the heat he found in the centre of a large tree was the effect of vitality. We can readily conceive, that if a thermometer is put into the centre of a large tree, and the air carefully excluded, that the mercury would be raised some few degrees above the freezing point, although the surrounding air might be several degrees below it; but, that this is an effect of vitality, I deny, on the following grounds:—The poplar was a young healthy tree, and every one knows it to be a quick growing tree; it must therefore abound with vitality: the bulb of the thermometer was placed as near to the centre of it as possible, and would have absorbed heat had there been any there developed. It may be asked, how we account for the mercury in the thermometer being raised in a large tree, and not in the small one? To this I reply, that the surrounding air does not absorb the heat from the centre of a large body sufficiently to reduce it to the freezing point of temperature. The thermometer may be placed the same distance under ground with the air carefully excluded, and with the same result. The farmer well knows this, who grows potatoes: his usual mode of preserving them from the frost is to clamp them in the field, which he does by throwing some earth out of a pit, putting in his potatoes, covering them with straw, and then throwing about a foot of earth over them. This is sufficient to protect them from a sharp winter's frost. If heat is vitality, we have as much right to insist upon the vitality of the earth, as they have upon the vitality of the tree. The confounders of vitality with heat will, perhaps, say, that the potatoes have vitality, and will not freeze. It is true they have vitality; but to say they will not freeze, is going a little too far. I shall not now dispute about this point, but find something else to answer my purpose; for we have a choice of many things.

It is a common practice, during a severe frost, to wrap pumps and water-pipes with hay-bands; and this, generally, answers the purpose of preventing the water from being frozen within: it must by these means be kept above the freezing point, although the air surrounding the pump may be several degrees below. It would be too ridiculous to say that vitality was in the hay-bands, which kept the water from freezing; but we have as much right to insist upon this as the confounders of heat with vitality have to insist that the heat of the tree arises from vitality. I repeat, then, that it was the bulk of the tree that protected the middle from the frost, and thus kept the thermometer above the freezing point, and not vitality.

Mr. Hunter, I believe, has somewhere stated, that he tried an experiment on the unimpregnated pullet's egg and the impregnated hen's egg. The latter he found to require three minutes longer to freeze than the former, in a freezing mixture prepared for the purpose. From this he drew his inference, that the vitality of the hen's egg resisted the frost the length of time stated. I believe it to be a hasty inference, and not a correct one: for, to prove the accuracy of the inference, he should have given us the specific gravity of each egg, and also the bulk or quantity of matter contained in each egg. I suspect that he has erred in this experiment, in the same manner as he did respecting the vitality of the tree resisting the cold and keeping the thermometer three degrees higher than the air surrounding the tree. It is known to most men that a hen's egg is usually bigger than a pullet's; and it must be obvious to every person, that, the greater the quantity of matter to be frozen in a freezing mixture, the greater length of time it will take to freeze: and it is thus that I account for the hen's egg taking three minutes longer to freeze than the pullet's, and I believe that vitality had nothing to do with it.

That heat is not vitality, is clear; for animal matter which is dead may be kept at a temperature constantly above that of living matter, yet all the functions of life will cease, and the heat, so far from keeping the substance alive, facilitates it much in its decomposition. If heat is not vitality, vitality cannot be heat; but heat may be an effect of vitality, and to a certain modification it is: our inquiries shall be directed how far vitality is connected with heat. All living matter is organized, and each organ has a certain function to perform. One of the principal functions of animation is breathing, or in some way having connexion with the atmospheric air, for vitality without this cannot be carried on; and this seems to be the chief origin of the heat of animals. Indeed, we see no other source of heat, but by the absorption of either air or water, either for animals or vegetables, and condensation of it when absorbed. The water, I believe, has but little to do with animal heat, and almost as little in vegetable; although it may, in some instances, operate a little upon vegetable matter, and raise its temperature, in the birch, the maple-tree, and some few others, in the month of March: but of this we are not certain; we only know that several gallons of liquor may be drawn from a tree of this description, and it is possible that condensation of the water absorbed by the roots may be going on quick enough to create heat faster than the surrounding air absorbs it. This is doubtful, as the specific gravity of the tree is less than that of water, and the supposed effect can, at most, happen only in one season of the year. Water, we will venture to say, is no source of heat to the fish above its own temperature, because condensation cannot go on to any extent without increasing the bulk of the fish inconsistent with its growth or its specific gravity, which would be so much increased that the fish would not swim, even with assistance of the air-bladder which it contains.

The law which all matter, with few exceptions, seems to obey respecting heat, is this:—When the space which it occupies is lessened, the density, and also its temperature, are increased. Heat has a propensity to find its equilibrium; it therefore radiates to the surrounding matter, until the body giving off heat finds the mean of its own temperature. To this general law there are some few exceptions, and water is one body of matter that deviates from this general rule to a certain extent; but we shall have occasion to speak of this hereafter. The heat of all animal bodies proceeds from food put into the stomach, or air taken into the lungs. Let us at present inquire how far heat can be attributed to the process of digestion, and afterwards to that of respiration. It is not my present purpose to run into a minute detail of digestion. When the food is put into the stomach, the gastric juice is secreted from the blood, and, as condensation takes place at the time of secretion, this is the source of a small quantity of heat, originating from food and air. As the process of digestion takes place, the contents of the stomach are decomposed; and, if recombined into less space, this may also be another source of heat. When digestion and absorption have taken place, and the food has been mixed and converted into blood, the glands and capillary vessels next take their part. If the new blood received from the food is decomposed and recombined by the glandular operation, and if the result of the combination fills less space than the new blood, then heat will also be given off; but all these can constitute but a small source of heat, and most probably would not generate heat sufficient to raise our bodies more than a very few degrees above the surrounding medium, and from this obvious reason,—the animals which eat the most are not always the hottest.

On inquiring how far respiration can be connected with the heat of animal bodies, we shall find that the heat of all animals is proportionate to the quantity of air consumed in their respiration. Winged insects, such as the bee, fly, &c. have their pulmonary apparatus most complete; they likewise consume more air in their respiration, in proportion to their size, than any other class of animals: the temperatures of their bodies are also the highest that are known to us. The feathered tribes are,

I believe, the next in order in their perfection as to their pulmonary apparatus, which even extends into their cylindrical bones. They consume more air, in proportion to their bulk, than the quadrupeds; their temperatures are also about ten degrees higher. The quadrupeds come next in order, as to the perfection of their pulmonary apparatus; and they are also next in order in the consumption of air in respiration, and in the heat of their bodies. On descending the line of creation to reptiles and fishes, we find that they do not consume enough air even to keep their heat above the surrounding medium, but are dependant thereon for the heat they possess: thus it is that toads and snakes, in our climate, make their escape in the autumn, and bury themselves some distance under ground. The snake, when dug from its hiding place in frosty weather, will hiss and seem half animated; but, in a short space of time, if it be exposed to the frosty air, it will become more torpid, and in a little time it will be frozen: which it has but little more power of resisting than the clod it lays upon.

There appear to be fish of two descriptions, cold and hot blooded; and there may be a middle class between the two,—as Mr. Hunter states that he found the stomach of a carp to be several degrees hotter than the water in which it swam. This I acknowledge to be a little in discordance with the foregoing arguments; but it is possible that this, and some other species of fish, may absorb air enough from the water to raise their temperature some few degrees; as carp are said to live out of water some weeks, and not only to live out of their element, but to get fat in this condition. That fish do absorb air to carry on vitality, is evident from the following observations:—They cannot exist under the recipient of an air-pump when the air is exhausted; they are also known sometimes to die when the water of a pond is frozen over, especially when there are no weeds, through which they in general have communication with the air; nor can they live in distilled water, or water that has been lately boiled, so as to drive off the air the water held in solution. The consumption of air in the gills of fish with one ventricle and one auricle, is small; so that the temperature of their bodies cannot be raised above the medium in which they swim. If a small portion of heat were to be generated in little fish, the surrounding water would absorb it much faster than the air, and would keep the temperature down to that of the medium surrounding them. The assertion of their resisting heat, and keeping their temperatures less than that of the surrounding medium, is truly ridiculous. It may be affirmed that there are no obvious possible means how this can take place, unless the fish possess the power of decomposing the water into its constituent gases.

We are now come to the cetaceous tribes of fish, which are called hot-blooded: they possess the double circulation and breathe with a pulmonary apparatus similar to that of quadrupeds; they inhabit a polar region, swim in a half-frozen ocean surrounded with huge masses of ice, breathing an atmosphere fifty degrees below zero: yet they are said to be of the same temperature as that of man, and their structure seems well calculated to generate and support this degree of heat. They possess an extensive pulmonary apparatus to generate heat, are of very great bulk, and their muscles are coated a foot thick with blubber, which is a bad conductor of heat.

With respect to the heat of unorganized bodies, it is known that, if a pound of ice at 32° be powdered and put into a pound of water heated to 172°, the whole quantity of ice and water will be reduced to 32°; consequently, 140 degrees of heat are lost, if we take the mean temperature. What becomes of this 140 degrees of heat,—is an enigma which has perplexed chemists for the last half century. Let us try to give some rational explanation of it. Water is said, at forty degrees, to gain its greatest specific gravity. Under this temperature it expands, and above this temperature it expands: however, it is more convenient for our argument to take it at 32°, the freezing point. When water is congealed, and becomes ice at 32°, it is expanded equal to 140 degrees; the water heated to 172° is expanded equal also to 140 degrees. In this case we see a double operation,—water expanded by heat, and water expanded by cold; and, when added together, the water at 172° gives off this 140 degrees of heat, and contracts as much as it was before expanded by heat. The ice receives this 140 degrees of heat, and also contracts as much as it was before expanded by cold. This is probable, as far as the thing is explicable, until we ascertain whether heat be matter or the property of matter.

*Leicester; January 20th,* 1821.

